# Atypical Scrapie Prions from Sheep and Lack of Disease in Transgenic Mice Overexpressing Human Prion Protein

**DOI:** 10.3201/eid1911.121341

**Published:** 2013-11

**Authors:** Jonathan D.F. Wadsworth, Susan Joiner, Jacqueline M. Linehan, Anne Balkema-Buschmann, John Spiropoulos, Marion M. Simmons, Peter C. Griffiths, Martin H. Groschup, James Hope, Sebastian Brandner, Emmanuel A. Asante, John Collinge

**Affiliations:** University College London, London, UK (J.D.F Wadsworth, S. Joiner, J.M. Linehan, S. Brandner, E.A. Asante, J. Collinge);; Federal Research Institute for Animal Health, Greifswald-Insel Riems, Germany (A. Anne Balkema-Buschmann, M.H. Groschup);; Animal Health and Veterinary Laboratories Agency, Addlestone, UK (J. Spiropoulos, M.M. Simmons, P.C. Griffiths, J. Hope)

**Keywords:** Atypical scrapie, bovine spongiform encephalopathy, BSE, Nor98 scrapie, prions, prion disease, prion protein, scrapie, sheep BSE, transgenic mice, transmissible spongiform encephalopathy (TSE), variant Creutzfeldt-Jakob disease, vCJD

## Abstract

Public and animal health controls to limit human exposure to animal prions are focused on bovine spongiform encephalopathy (BSE), but other prion strains in ruminants may also have zoonotic potential. One example is atypical/Nor98 scrapie, which evaded statutory diagnostic methods worldwide until the early 2000s. To investigate whether sheep infected with scrapie prions could be another source of infection, we inoculated transgenic mice that overexpressed human prion protein with brain tissue from sheep with natural field cases of classical and atypical scrapie, sheep with experimental BSE, and cattle with BSE. We found that these mice were susceptible to BSE prions, but disease did not develop after prolonged postinoculation periods when mice were inoculated with classical or atypical scrapie prions. These data are consistent with the conclusion that prion disease is less likely to develop in humans after exposure to naturally occurring prions of sheep than after exposure to epizootic BSE prions of ruminants.

Bovine spongiform encephalopathy (BSE) is the transmissible spongiform encephalopathy (TSE) or prion disease of domestic cattle. The BSE prion is an epizootic agent and causes variant Creutzfeldt-Jakob disease (vCJD) in humans after dietary exposure (*1*–*4*). Because the time lag between exposure and development of vCJD may be decades, uncertainty about the extent of the pathogenicity of BSE for humans continues (*5*), and subclinical forms of infection may exist (*6*,*7*). A recent immunohistochemical study that estimated prevalence of prion infection in the UK population by screening samples from surgically removed appendixes found 1 in 2,000 persons were positive for the disease-associated form of the prion protein (PrP) (*8*). Similar uncertainty exists in our understanding of scrapie, the TSE of small ruminants, which has been heightened in recent years by finding BSE in goats (*9*,*10*), the possibility of BSE in sheep (*11*), and the discovery of atypical scrapie (*12*,*13*), a form of small-ruminant TSE, which had evaded statutory diagnosis until the early 2000s.

Recent analysis of surveillance data of TSEs in small ruminants in Great Britain, collected over the past 10 years, has demonstrated a dramatic decrease (up to 90%) in number of confirmed cases of classical scrapie in the national flock. However, atypical scrapie continues to affect sheep bred for their relative resistance to the classical form of this prion disease, and the proportion of sheep with resistant genotypes in the national flock is likely to have increased over the past decade because of the National Scrapie Plan for Great Britain. This increase has rekindled speculation that atypical scrapie in small ruminants might be a source of human prion disease (*11*). Although atypical scrapie has been discovered retrospectively in 2 UK sheep culled in 1987 and 1989 (*14*,*15*), the level and duration of human exposure to atypical scrapie prions are unknown, and this lack of knowledge confounds a cause-and-effect investigation of epidemiologic links between this animal disease and some form of CJD (*11*).

Over the past 2 decades, surrogate methods have been developed to assess the relative pathogenicity of animal prions for humans. One approach involves the experimental transmission of disease by inoculating homogenized brain tissue from affected animals into transgenic mice that are overexpressing 1 of the 2 common polymorphic forms of the human PrP (either methionine or valine at residue 129) on a mouse PrP null background (*16*). Such transgenic mice are fully susceptible to infection with human prions (*16*) and, to a lesser extent, cattle and ovine BSE prions (*2*,*4*,*17*), but appear resistant to chronic wasting disease prions from cervids (*18*–*20*). In this study, we inoculated transgenic mice that overexpressed human PrP with brain tissue from field sheep with natural cases of classical and atypical scrapie, sheep with serially-passaged experimental BSE, and cattle with BSE to assess the pathogenicity of natural scrapie prions relative to that of the known epizootic TSE agent, the cattle BSE prion strain.

## Materials and Methods

### Ovine and Bovine Prion Sources

From Great Britain’s Animal Health and Veterinary Laboratories Agency (AHVLA), we obtained 10% (w/v) brain homogenates prepared in sterile saline from sheep with neuropathologically confirmed prion disease and demonstrated ability to transmit prion disease to transgenic mice expressing ovine PrP or to wild-type mice (Table 1). We obtained scrapie-infected sheep brain from the Friedrich-Loeffler-Institut (Federal Research Institute for Animal Health, Germany) under a license granted by Department for Environment, Food and Rural Affairs, according to the terms of the Importation of Animal Pathogens Order 1980. Brain samples from sheep with neuropathologically confirmed cases of classical and atypical scrapie were prepared as 10% (w/v) homogenates in sterile Dulbecco phosphate-buffered saline lacking Ca^2+^ and Mg^2+^ ions (D-PBS) by extrusion through syringe needles of decreasing diameter. Brains from cattle with neuropathologically confirmed cases of BSE (collected specifically for transmission studies in the early 1990s) were provided by the UK Central Veterinary Laboratory (now AHVLA). We used 10% (w/v) homogenates prepared from the brainstems of 5 cattle with natural BSE to generate pooled inocula, designated I038, which was previously shown to transmit prion disease to wild-type FVB/N and C57Bl/6 mice, and to transgenic mice overexpressing human PrP (*2*,*4*,*23*,*24*). All experimental procedures involving ovine or bovine prions were carried out in a microbiological containment level 3 facility with strict adherence to safety protocols.

**Table 1:** Details of prion sources for ovine inocula*

Source code	Brain region	Prion agent	Ovine PrP genotype‡		Transmission data†	
					Attack rate (incubation period)	Reference
AHVLA/SE 1919/0077	Cerebral cortex	Classical scrapie	VRQ/VRQ		tg338 mice§; 16/16 (64 ± 2 d)	(*21*); code SE 1848/0005
AHVLA/SE 1919/0080	Cerebral cortex	Classical scrapie	ARQ/ARQ		tg338 mice§ 12/13 (155 ± 4 d)	(*21*); code SE 1848/0008
FLI 1/06	Caudal medulla	Classical scrapie	ARQ/ARQ		ND	NA
FLI 83/04	Caudal medulla	Classical scrapie	ARQ/ARQ		ND	NA
FLI 107/04	Caudal medulla	Classical scrapie	ARQ/ARQ		ND	NA
AHVLA/SE 1850/0001	Caudal medulla	Atypical scrapie	AHQ/AHQ		tg338 mice§; 19/20 (210 ± 3 d)	(*22*); code 1
AHVLA/SE 1850/0009	Caudal medulla	Aypical scrapie	ARR/ARR		tg338 mice§; 19/19 (231 ± 6 d)	(*22*); code 9
FLI S7/06	Caudal medulla	Atypical scrapie	AHQ/ARQ		ND	NA
FLI 14/06	Caudal medulla	Atypical scrapie	ARR/ARR		ND	NA
FLI 26/06	Caudal medulla	Atypical scrapie	AHQ/ARQ		ND	NA
AHVLA/SE 1929/0877	Caudal medulla	Ovine BSE	ARQ/ARQ		RIII mice; 16/19 (422 ± 19 d)¶	Unpub.
AHVLA/SE1945/0032	Rostral medulla	2nd Passage ovine BSE	ARQ/ARQ		RIII mice; 18/20 (356 ± 9 d)¶	Unpub.

*PrP, prion protein; AHVLA, Animal Health and Veterinary Laboratories Agency; ND, not done; NA, not applicable; FLI, Friedrich-Loeffler-Institut; BSE, bovine spongiform encephalopathy; Unpub., unpublished.  †Reports attack rate (the no. of infected mice as a proportion of the no. of inoculated mice) and mean incubation period in days ± SEM or SD and the reference in which the data were first published with original inocula code.  ‡Ovine PrP codon 136,154,171 genotype. §Transgenic for the ovine *Prnp* VRQ allele on a mouse *Prnp*^0/0^ background. Overexpression 8- to 10-fold of normal ovine brain. ¶Mean incubation period in days ± SEM.

### Transgenic Mice

Transgenic mice homozygous for a human PrP 129V transgene array and murine PrP null alleles (*Prnp^o/o^*), designated Tg(HuPrP129V^+/+^ *Prnp^o/o^*)-152 mice (129VV Tg152 mice), or homozygous for a human PrP 129M transgene array and murine PrP null alleles (*Prnp^o/o^*), designated Tg(HuPrP129M^+/+^ *Prnp^o/o^*)-35 mice (129MM Tg35 mice), have been described (*1*,*2*,*4*,*24*–*26*). Both lines of mice were used to generate FVB/N-HuPrP^+/+^ *Prnp*^o/o^ congenic lines by backcrossing to FVB/N mice for 10 generations, followed by genetic testing (Charles River UK, Ltd., Margate, UK) by using 84 FVB-specific PCR microsatellite markers covering 19 chromosomes at ≈20-cM intervals, to select breeding pairs positive for 100% of the FVB-specific markers. Selected congenic pairs were interbred to remove the endogenous murine PrP gene and to establish homozygosity of the human PrP transgene array. The resulting congenic lines, designated 129MM Tg35c and 129VV Tg152c, overexpress human PrP in brain at levels of 2× and 6× that of pooled human brain, respectively.

### Transmission Studies

Work with animals was performed under a license granted by the UK Home Office and conformed to institutional guidelines of the University College London and ARRIVE (Animal Research: Reporting In Vivo Experiments guidelines of The National Centre for the Replacement, Refinement and Reduction of Animals in Research). Brain homogenates (10% w/v) were diluted to 1% (w/v) in sterile D-PBS and passed through a 25-gauge needle. Each mouse was inoculated with 30-μL of 1% (w/v) brain homogenate because this avoids excessive animal losses within the first 48 hours postinoculation (*4*). Brain homogenates from prion-infected sheep were inoculated intracerebrally into groups of 20 transgenic mice that overexpressed human PrP. Thereafter, mice were examined daily and killed if they were exhibiting signs of distress or once a diagnosis of clinical prion disease was established (*4*,*24*,*25*). Clinical diagnosis can be confounded by nonspecific conditions that develop in mice as they age, and the mean lifespans of different lines of transgenic mice and the onset of aging artifacts vary greatly. On the basis of experience, we have limited these confounding effects by electively culling mice after postinoculation periods of >600 days. Notably, this also helps reduce the number of mice that die of old age, in which brain tissue can undergo autolytic deterioration that impairs immunohistochemical (IHC) analyses. At post-mortem, brains from inoculated mice were removed and divided sagittally, with half of the samples frozen and half fixed in formol-saline, and analyzed for abnormal PrP accumulation by IHC and immunoblotting.

### Neuropathologic and Immunohistochemical Analyses

Brain fixed in 10% buffered formol-saline was immersed in 98% formic acid for 1 hour and embedded in paraffin wax. Serial sections (4-μm thick) were pretreated by boiling for 10 min in a low ionic strength buffer (2.1 mmol/L Tris, 1.3 mmol/L EDTA, 1.1 mmol/L sodium citrate, pH 7.8) before exposure to 98% formic acid for 5 min. Abnormal PrP accumulation was examined by using monoclonal antibody ICSM 35 against PrP (D-Gen Ltd., London, UK) on an automated IHC staining machine (Ventana Medical Systems, Inc., Tucson, AZ, USA) by using proprietary secondary detection reagents (Ventana Medical Systems, Inc.) before development with 3′3-diaminobenzedine tetrachloride as the chromogen (*27*). Conventional methods were used for Harris hematoxylin and eosin staining. Appropriate positive and negative controls were used throughout. Photographs were taken on an ImageView digital camera and composed with Adobe Photoshop (Adobe Systems, San Jose, CA, USA).

### Immunoblotting

Proteinase K (PK) digestion (50 or 100 μg/mL final protease concentration, 1 hour, 37°C), electrophoresis, and immunoblotting of 10% (w/v) transgenic mouse brain homogenates or 10% (w/v) brain homogenates from sheep with classical scrapie (prepared in D-PBS) were performed as described (*27*,*28*). Human PrP or ovine PrP was detected by using monoclonal antibodies 3F4 (*29*) or ICSM 35 against PrP (D-Gen Ltd.), respectively. Mouse brain homogenates found negative for disease-related PrP (PrP^Sc^) after analysis of 10 μL 10% (w/v) brain homogenate were reanalyzed by sodium phosphotungstic acid (NaPTA) precipitation of PrP^Sc^ (*30*) from 250 μL of 10% (w/v) brain homogenate as described (*30*).

Atypical scrapie sheep brain was analyzed by using the procedure of Gretzschel et al. (*31*,*32*) with modifications. In brief, 200 μL of 10% (w/v) brain homogenate in D-PBS was centrifuged at 500 × *g* for 5 min, after which the supernatant was discarded, and the pellet was resuspended to 100 μL final volume with D-PBS, followed by the addition of 100 μL 4% (w/v) sodium lauroylsarcosine (sarkosyl) in D-PBS. After incubation at 37°C for 30 min with constant agitation and centrifugation at 500 × *g* for 5 min, 150 μL of the supernatant was transferred to a new tube. The supernatant fraction was treated with 2 μL of Benzonase (Benzon nuclease purity 1; 25 U/μL; Merck, Nottingham, UK) for 30 min at 37°C with agitation and adjusted to a final concentration of 50 μg/mL PK (by adding 8 μL of a 1 mg/mL PK stock solution) and incubated at 37°C for 60 min with agitation. Samples were treated with 4 μL 100 mmol/L 4-(2-aminoethyl)-benzene sulfonyl fluoride, heated at 100°C for 5 min, adjusted with an equal volume of 2% (w/v) sarkosyl in D-PBS and 3 μL of Benzonase; they were then incubated for 30 min at 37°C with agitation before addition of 4% (w/v) NaPTA containing 170 mmol/L MgCl_2_, pH 7.4, to give a final concentration in the sample of 0.3% (w/v) NaPTA. After incubation for 60 min at 37°C, with constant agitation, samples were centrifuged at 16,100 × *g* for 30 min, and the supernatant fraction was discarded. The pellet fraction was resuspended to a final volume of 10 μL in D-PBS containing 0.1% (w/v) sarkosyl and analyzed by electrophoresis, immunoblotting, and high sensitivity chemiluminescence (*27*,*28*), using monoclonal antibody ICSM 35 against PrP to detect ovine PrP.

## Results

### Scrapie Prions from Sheep and Lack of Disease in Transgenic Mice 

**Figure 1:**
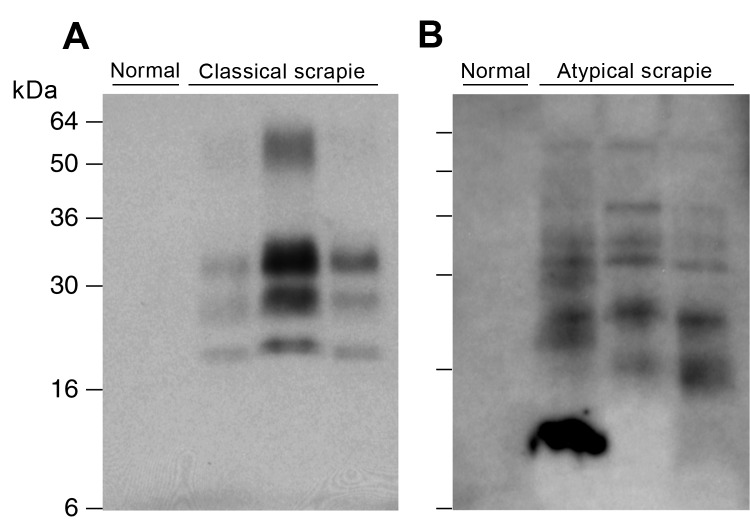
Detection of disease-related prion protein (PrP^Sc^) in brains of sheep with field cases of classical and atypical scrapie, Germany. Both panels show immunoblots of proteinase K–digested brain homogenate analyzed by enhanced chemiluminescence with monoclonal antibody ICSM 35 against PrP. Samples in panel A are from 10 μL 10% (w/v) brain homogenate after direct digestion with protease. Samples in panel B are derived after processing 200 μL10% (w/v) brain homogenate as described in Materials and Methods. A) From left, normal sheep brain compared to classical scrapie sheep brain samples FLI 1/06, FLI 83/04 and FLI 107/04. B) From left, normal sheep brain compared to atypical sheep scrapie brain samples FLI S7/06, FLI 14/06 and FLI 26/06.

We examined classical and atypical scrapie sheep brain homogenates from UK field cases (AHVLA) that contain PK-resistant ovine PrP^Sc^ and efficiently transmitted clinical prion disease to transgenic mice expressing ovine PrP (*21*,*22*) (Table 1), together with a series of PK-resistant PrP-positive brain homogenates, from sheep in Germany with field cases of classical and atypical scrapie (Figure 1). All natural brain isolates examined produced no clinical prion disease or biochemical or histopathologic evidence for subclinical prion infection in transgenic mice that overexpressed human PrP after postinoculation intervals of >600 days (Table 2). 

**Table 2:** Survival times of transgenic human PrP mice after inoculation of ovine prions*

Source code	Prion agent	Transmission data				
		129MM Tg35c mice			129VV Tg152c mice	
		Attack rate†	Survival, d‡		Attack rate†	Survival, d‡
AHVLA/SE 1919/0077	Classical scrapie	0/20	551, 551, 583, 615–666 (17)		0/16	301, 344, 344, 364, 386, 428, 475, 519, 540, 543, 600–601 (6)
AHVLA/SE 1919/0080	Classical scrapie	0/19	580, 586, 586, 620–666 (16)		0/14	211, 336, 364, 364, 379, 519, 601–602 (8)
FLI 1/06	Classical scrapie	0/15	426, 475, 628–728 (13)		0/17	364, 497, 498, 517, 547, 559, 571, 595, 603–673 (9)
FLI 83/04	Classical scrapie	0/15	270, 307, 311, 335, 349, 353, 635–672 (9)		0/16	227, 300, 335, 440, 479, 510, 600–650 (10)
FLI 107/04	Classical scrapie	0/17	382, 382, 459, 573, 574, 578, 606–636 (11*)*		0/13	227, 228, 476, 606–706 (10)
AHVLA/SE 1850/0001	Atypical scrapie	0/18	213, 332, 437, 537, 537, 621–656 (13)		0/18	255, 318, 385, 397, 402, 403, 452, 453, 493, 518, 528, 538, 543, 552, 633–647 (4)
AHVLA/SE 1850/0009	Atypical scrapie	0/18	440, 606–635 (17)		0/15	293, 334, 403, 404, 419, 420, 426, 444, 584, 637–651 (6)
FLI S7/06	Atypical scrapie	0/16	498, 610–659 (15)		0/14	539, 545, 630–673 (12)
FLI 14/06	Atypical scrapie	0/18	538, 540, 545, 572, 601–728 (14)		0/15	313, 363, 489, 510, 592, 602–673 (10)
FLI 26/06	Atypical scrapie	0/14	547, 553, 643–659 (12)		0/14	435, 446, 554, 571, 608–673 (10)
AHVLA/SE 1929/0877	Ovine BSE	0/16	315, 316, 348, 459, 557, 581, 620–659 (10)		0/18	358, 363, 369, 382, 385, 440, 468, 476, 532, 550, 574, 600–602 (7*)*
AHVLA/SE 1945/0032	2nd Passage ovine BSE	1/19	337, 337, 434, 472, 517, 524, 616–661 (13)		0/17	331, 331, 381, 386, 388, 388, 525, 527, 542, 562, 603–608 (7)

*PrP, prion protein; AHVLA, Animal Health and Veterinary Laboratories Agency; FLI, Friedrich-Loeffler-Institut; BSE, bovine spongiform encephalopathy. †All mice were inoculated with 30 μL of 1% (w/v) brain homogenate. Attack rate is defined as the total number of clinically affected and subclinically infected mice as a proportion of the number of inoculated mice. Subclinical prion infection was assessed by immunohistochemical examination of brain for abnormal PrP deposition and for recipients of AHVLA inocula by sodium phosphotungstic acid precipitation of 250 μL 10% brain homogenate and analysis for PrP^Sc^ by proteinase K digestion and immunoblotting. ‡The interval between inoculation and culling because of intercurrent illness, senescence, or termination of the experiment in days. Death dates of individual mice are shown together with the range for mice surviving beyond 600 d with the number of mice in this range shown in parentheses. Mice culled with postinoculation periods of ≤200 d due to intercurrent illness (all confirmed negative for prion infection) were not included in calculating attack rates.

Consistent with the inability of IHC or high sensitivity immunoblotting to detect pathologic PrP in the brains of inoculated mice, neuropathologic examination of the brain showed no difference in spongiform change or gliosis from that observed in the brains of age-matched control mice (data not shown). From these findings, we conclude that both methionine and valine residue 129 variants of human PrP are refractory to pathologic conversion by these ovine prion strains in transgenic mice.

### Transmission of Cattle BSE Prions to Transgenic Mice 

Brain isolates from sheep with classical and atypical scrapie (including those with demonstrated prion infectivity in transgenic mice expressing ovine PrP) did not transmit prion disease to transgenic mice that were overexpressing human PrP. This fact contrasts markedly with the known susceptibility of these mice to transmission of multiple cattle BSE isolates (*2*,*4*,*24*,*25*) as well as to transmission of a wide range of human-acquired prion diseases (including kuru and vCJD) and sporadic prion disease isolates (*2*,*4*,*24*–*26*).

**Figure 2:**
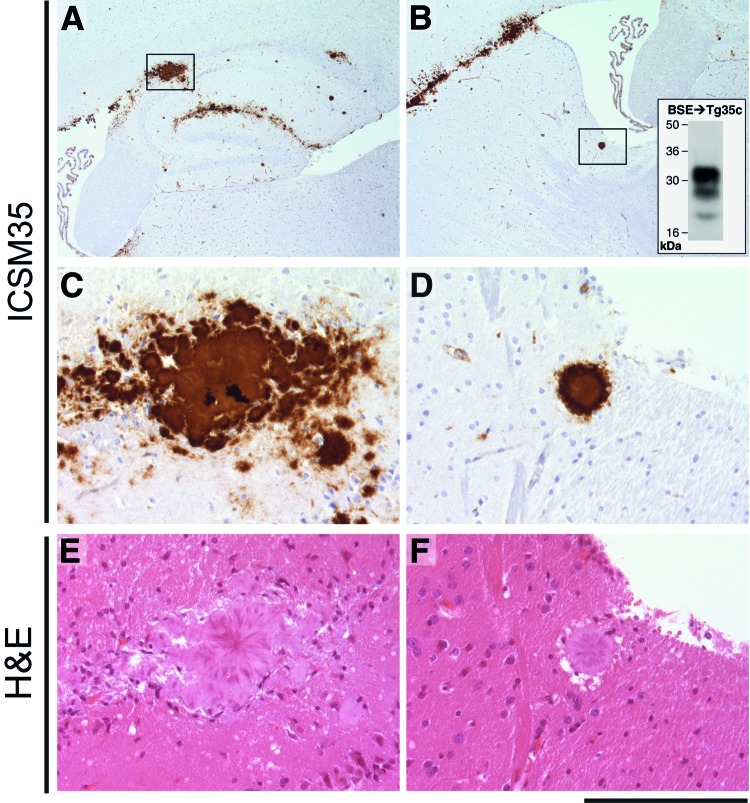
Immunohistochemical analysis of cattle bovine spongiform encephalopathy (BSE) prion–infected 129MM Tg35c mouse brain. Hippocampal region (A) and striatum (B) from a transgenic 129MM Tg35c mouse with subclinical prion infection culled 700 days after inoculation with cattle BSE prion inoculum I038. Panels A–D show abnormal prion protein (PrP) immunoreactivity stained with monoclonal antibody ICSM35 against PrP. Panels E and F show hematoxylin and eosin–stained sections. Boxed regions in panels A and B are shown at higher power magnification in panels C and E, and D and F, respectively. The inset in panel B shows an immunoblot in which monoclonal antibody 3F4 against PrP was used, which demonstrates type 4 PrP^Sc^ in 10 μL of PK-digested 10% (w/v) brain homogenate prepared from the contralateral side of the same brain. Scale bar indicates 1.2 mm for panels A and B, 160 μm for panels C–F.

Concomitant with the current study, and as part of a separate experiment, we inoculated 129MM Tg35c mice intracerebrally with cattle BSE isolate I038. This BSE isolate has previously been shown to be transmissible to the parent 129MM Tg35 transgenic line, producing an attack rate of 8/20 inoculated mice (*4*) (Table 3). Affected 129MM Tg35 mice in these transmissions were culled (because of intercurrent illness or clinical prion disease) within 600 days of inoculation (Table 3) and demonstrated the presence of abnormal PrP in brain by IHC and immunoblotting (*4*). In 129MM Tg35c mice, cattle BSE isolate I038 produced an attack rate of 5/12 in intracerebrally inoculated mice (Table 3). Infection was characterized by the detection of abnormal PrP by IHC (Figure 2, panels A, B), which included large amorphous PrP deposits (Figure 2, panels C, E) and florid PrP plaques (Figure 2, panels D, F), and the detection of type 4 PrP^Sc^ in brain homogenate by immunoblotting (Figure 2, panel B inset). Intercurrent illness before 600 days postinoculation was seen in only one 129MM Tg35c mouse, with the remaining mice in the group (11/12) culled 611–853 days postinoculation (Table 3). Although most mice survived >600 days after inoculation, the attack rate of cattle BSE isolate I038 in 129MM Tg35c mice remained the same as observed in the parental 129MM Tg35 mouse line with ≈40% of inoculated mice becoming infected (Table 3). In addition, we found that 129MM Tg35 and 129MM Tg35c mice showed equivalent susceptibilities (100% attack rates) to vCJD or classical CJD prions (Table 3).

**Table 3:** Survival times of transgenic human PrP 129MM mice after inoculation of cattle BSE, vCJD, or classical CJD prions*

			Transmission data				
			129MM Tg35 mice			129MM Tg35c mice	
Inocula source code	Prion agent		Attack rate†	Survival, d‡		Attack rate†	Survival, d‡
MRC I038	Cattle BSE		8/20	263,316,333,344,389,400, 411, 468 488, 578, 593, 627–876 (9)§		5/12	484, 611–853 (11*)¶*
MRC I344	vCJD		7/7	342, 432, 487,516, 650–726 (3)		12/12	378, 447, 558, 586, 628–793 (8)#
MRC I026	Classical CJD**		7/7	215,222,222,222,222,228,228††		9/9	223,223,223,223,226,226,226, 227,227††

*PrP, prion protein; MRC, Medical Research Council; BSE, bovine spongiform encephalopathy; vCJD, variant Creutzfeldt-Jakob disease; CJD, Creutzfeldt-Jakob disease. †All mice were inoculated with 30 μL of 1% (w/v) brain homogenate. Attack rate is defined as the total number of both clinically affected and subclinically infected mice as a proportion of the number of inoculated mice. Subclinical prion infection was assessed by immunohistochemical examination of brain for abnormal PrP deposition and analysis of 10% brain homogenate for disease-related PrP (PrP^Sc^)by proteinase K digestion and immunoblotting. ‡The interval between inoculation and culling because of intercurrent illness, clinical prion disease, senescence, or termination of the experiment in days. Death dates of individual mice are shown together with the range for mice surviving beyond 600 d with the number of mice in this range shown in parentheses. §Affected mice were culled at 316, 333, 344,389,400, 468, 578, and 593 d postinoculation. Mice culled at 344 and 468 d had clinical prion disease. ¶Affected mice were culled at 700, 720, 798, 817 and 853 d post-inoculation. The mouse culled at 720 d had clinical prion disease. #Two mice with clinical prion disease were culled at 558 and 749 d. **Iatrogenic CJD (dura mater), *PRNP* codon 129 methionine homozygous with type 2 PrP^Sc^ by the London classification (*33*). ††All mice had clinical prion disease.

### Experimental Ovine BSE in Transgenic Mice Expressing Human PrP 129 Methionine

**Figure 3:**
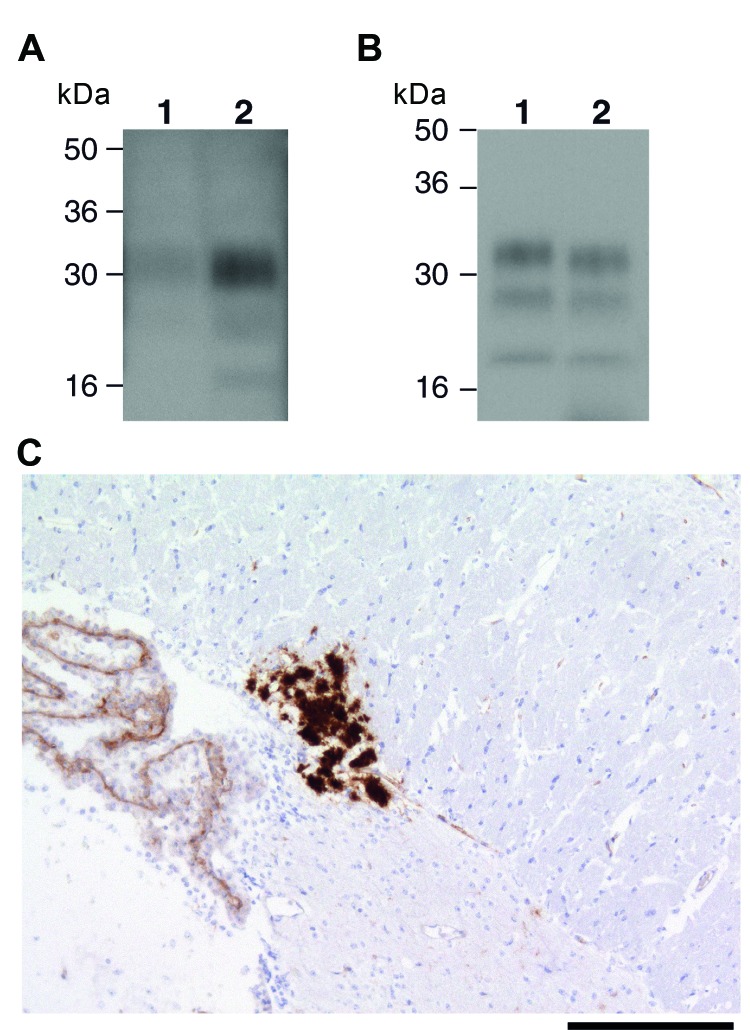
Ovine bovine spongiform encephalopathy (BSE) prion transmission to a 129MM Tg35c mouse. Panel A shows immunoblot detection of disease-related prion protein (PrP^Sc^) in 10 μL of proteinase K (PK)–digested 10% (w/v) brain homogenates from ovine BSE (SE 1929/0877) (lane 1) and secondary passage ovine BSE (SE1945/0032) (lane 2) using monoclonal antibody ICSM35 against prion protein (PrP). Panel B shows type 4 PrP^Sc^ in 1 μL of PK-digested 10% (w/v) vCJD brain homogenate (lane 1) in comparison to PrP^Sc^ in 20 μL of PK-digested 10% (w/v) brain homogenate from a 129MMTg35c mouse with subclinical prion infection that was culled 661 days after inoculation with secondary passage ovine BSE inoculum SE1945/0032 (lane 2). Panel C shows abnormal PrP immunoreactivity stained with monoclonal antibody ICSM35 against PrP in the corpus callosum of the ovine BSE–affected 129MM Tg35c mouse brain. Scale bar indicates 165 μm.

Recently, 2 studies have concluded that experimental sheep BSE prions may propagate more efficiently than cattle BSE prions in transgenic mice that express human PrP 129 methionine (*17*,*34*). One of these studies convincingly established that sheep and goat BSE prions transmitted a molecular and neuropathologic phenotype congruent with transmission of vCJD prions in the same mice (*17*). These data strongly suggest that small ruminant BSE prions could act as causal agents of vCJD (*17*). In this study, we also examined the transmission properties of 2 experimental sheep BSE brain isolates derived from the primary transmission and secondary passage of cattle BSE in sheep. These AHVLA isolates were provided as brain homogenates that contained PK-resistant ovine PrP (Figure 3, panel A) and had known ability to transmit clinical prion disease to wild-type RIII mice (Table 1).

In the transgenic mice expressing human PrP, clinical prion disease was not produced by either of the 2 experimental sheep BSE isolates after postinoculation intervals >600 days (Table 2). Examination of brain from these inoculated mice by IHC and immunoblotting, after NaPTA precipitation of brain homogenate, showed that only a single 129MM Tg35c recipient of the secondary passage ovine BSE isolate had evidence of subclinical prion infection (Tables 1, 2; Figure 3). This mouse was culled 661 days postinoculation when the experiment was terminated. PrP^Sc^ was detectable in the brain of this transgenic mouse without requirement for NaPTA precipitation for detection and appeared similar (but not identical) to type 4 PrP^Sc^ seen in vCJD brain (Figure 3, panel B). Florid PrP plaques were not observed, and abundant PrP deposits were restricted to the corpus callosum (Figure 3, panel C), accompanied by occasional punctate PrP deposits in the cortex and sparse diffuse PrP deposits in the thalamus and hypothalamus (data not shown). Secondary passages of this isolate in additional human PrP–expressing transgenic mice and wild-type FVB/N mice have been initiated to comprehensively define prion strain type.

Why the efficiency of transmission of experimental sheep BSE prions to 129MM Tg35c mice is low compared with that reported in different lines of human PrP 129 methionine–expressing mice (*17*,*34*) is unclear. One possible reason may simply relate to the prion titers in the inocula. Plinston et al. reported that 2 different inocula prepared from the same experimental sheep BSE brain had markedly different transmission efficiencies to gene-targeted mice expressing human PrP 129 methionine at endogenous levels (*34*). However, all AHVLA ovine prion isolates used in this study were chosen because they produced short survival periods and high attack rates in either ovine PrP transgenic mice or wild type mice (Table 1). Therefore, other possibilities must also be considered. In particular, studies involving different laboratories use different lines of genetically modified mice. Variation in genetic background and differences in PrP expression levels are known to influence host susceptibility to prion infection (*16*).

## Discussion

In this study, we have shown that disease does not develop in transgenic mice overexpressing human PrP when mice are inoculated with ovine prions from sheep with natural cases of classical scrapie and atypical scrapie from Great Britain and Germany. These transgenic mice are susceptible to infection, and clinical disease develops when mice are challenged with brain tissue from cattle affected by classical BSE (*2*,*4*,*24*,*25*) or brain tissue from humans affected by classical (sporadic and iatrogenic) CJD, kuru, or vCJD (*2*,*4*,*24*–*26*). Therefore, this suggests that the transmission barrier associated with the interaction of human PrP and the prion strain causing epizootic BSE in cattle is lower than that associated with the prion strain causing atypical scrapie in sheep. Serial, blind passage of brain homogenates from “negative” challenged mice from this experiment into other lines of transgenic mice expressing either human PrP or ovine PrP will now be required to determine whether this transmission barrier is absolute.

Our findings complement those of other recent studies that have investigated the zoonotic potential of ruminant prion strains using other lines of human PrP–expressing mice. Gene-targeted human PrP–expressing mice have been shown to be resistant to infection with classical and atypical scrapie prions from sheep (*34*,*35*) and BSE prions from cattle (*36*) but are susceptible to infection with BSE prions from sheep (*34*). Transgenic mice with 6-fold overexpression of human PrP 129 methionine are susceptible to infection with cattle BSE prions but show greater susceptibility to ovine and caprine BSE prions (*17*).

Although we found evidence for transmission of experimental ovine BSE to transgenic mice expressing human PrP 129 methionine, the relative attack rate was lower than observed in the other lines of mice (*17*,*34*). The reasons underlying this are not clear but may relate to differences in the prion isolates themselves or differences in the various lines of mice. To definitively investigate interlaboratory differences in the apparent behavior of ovine BSE prions and reach a consensus, a panel of ovine prion inocula would need to formally undergo endpoint titration across the different lines of humanized mice and also in ovine PrP–expressing transgenic mice.

No strain variation has been found so far in the transmission, biochemical, or histopathologic characteristics of atypical scrapie prions (*22*,*37*), and so inferences from the present study are not confounded by sampling or strain considerations. This is not so for cases of classical scrapie and, although our findings on atypical scrapie prions indicate that the zoonotic potential of this ovine prion strain is lower than for ruminant BSE prions, further transmission studies using a wider variety of field cases of classical scrapie are required to provide further reassurance of the low or negligible zoonotic potential of all sheep prions. Examining extraneural tissues (in particular, the spleen) in ovine prion-challenged mice will be critical because recent findings have shown that cross-species prion transmission efficacy can exhibit a dramatic tissue-dependence in the same host (*38*).
